# Transfer learning identifies bacterial signatures for cross‐regional diagnosis of type 2 diabetes and enable stage‐sensitive dietary fiber intervention

**DOI:** 10.1002/imo2.70021

**Published:** 2025-05-04

**Authors:** Qunye Zhang, Nan Wang, Fanghua Zhang, Bin Chen, Yihui Wang, Zhongchao Wang, Changying Zhao, Chuandi Jin, Dashuang Sheng, Kaile Yue, Daifeng Jiang, Liaomei Gao, Haohong Zhang, Zixin Kang, Mingyue Cheng, Xiaoli Ma, Haiyan Wang, Dongming Hu, Jun Wang, Yuantao Liu, Chenhong Zhou, Minxiu Yao, Guoping Zhao, Yangang Wang, Zhe Wang, Kang Ning, Lei Zhang

**Affiliations:** ^1^ Microbiome‐X, School of Public Health; Department of Cardiology, Qilu Hospital; Cheeloo College of Medicine Shandong University Jinan China; ^2^ Key Laboratory of Molecular Biophysics of the Ministry of Education, Hubei Key Laboratory of Bioinformatics and Molecular‐Imaging, Center of AI Biology, Department of Bioinformatics and Systems Biology, College of Life Science and Technology Huazhong University of Science and Technology Wuhan China; ^3^ Department of Endocrinology, Qingdao Central Hospital University of Health and Rehabilitation Sciences (Qingdao Central Hospital) Qingdao China; ^4^ Qingdao University Medical Group: Department of Endocrinology and Metabolism, The Affiliated Hospital of Qingdao University; Department of Endocrinology and Metabolism, The Third People's Hospital of Qingdao Qingdao China; ^5^ Shandong Academy of Sciences Science and Technology Services Platform (Shandong Academy of Sciences Foreign Students Pioneer Park) Jinan China; ^6^ Shandong Provincial Maternal and Child Health Care Hospital Affiliated to Qingdao University Jinan China; ^7^ Department of Endocrinology Qingdao Municipal Hospital (Group) Qingdao China; ^8^ Department of Endocrinology The Eighth People's Hospital of Qingdao Qingdao China; ^9^ Department of Endocrinology Qilu Hospital of Shandong University (Qingdao) Qingdao China; ^10^ State Key Laboratory of Microbial Technology Shandong University Qingdao China; ^11^ CAS Key Laboratory of Computational Biology, Bio‐Med Big Data Center, Shanghai Institute of Nutrition and Health University of Chinese Academy of Sciences, Chinese Academy of Sciences Shanghai China; ^12^ Department of Endocrinology & Geriatrics Shandong Provincial Hospital Affiliated to Shandong First Medical University Jinan China

## Abstract

DeepMicroFinder is a deep learning framework designed to update the existing disease diagnosis model to generate a transfer model by leveraging region‐specific microbiome datasets and transfer learning approach. This framework effectively overcomes the limitation of regional effects in the gut microbiome, enabling accurate cross‐regional disease detection. Microbial markers related to type 2 diabetes (T2D) were identified by DeepMicroFinder, and subsequently validated in independent T2D cohorts undergoing dietary fiber interventions.

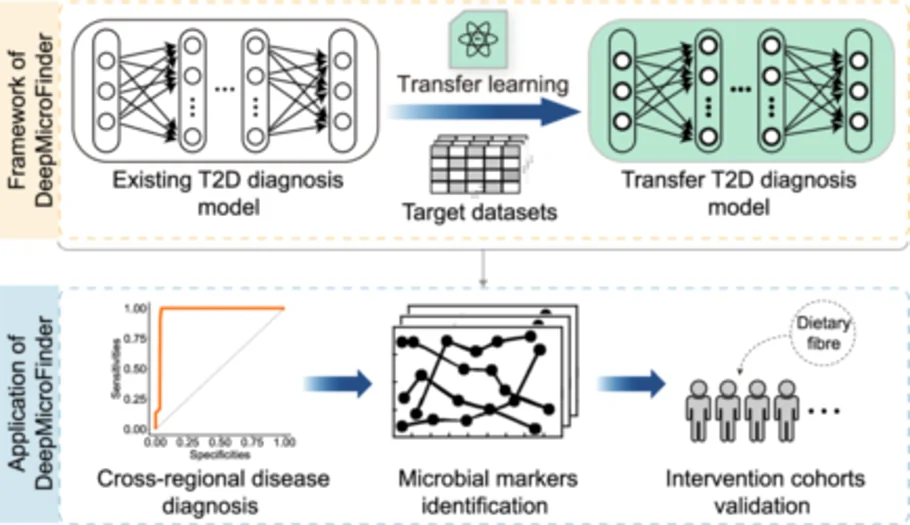

To the editor,

Type 2 diabetes (T2D) is a chronic metabolic disease characterized by hyperglycemia, insulin resistance, and relative insulin deficiency, influenced by factors such as genetics, diet, and medications [1, 2]. The gut microbes contribute to the human body's nutrient metabolism, immunity, and disease development [3]. Studies have shown that gut microbes' composition and function, as well as their related metabolites, are correlated with diabetic phenotypes like hyperglycemia and insulin resistance [4]. However, regional variations in gut microbes limit the accuracy of cross‐regional T2D diagnosis using current machine learning methods, and microbial markers identified in one region may lack universality in others [5]. Additionally, dietary fiber intervention (DFI) is a common clinical therapy for T2D [6], but its mechanisms remain inconsistent. To address these issues, we proposed a deep learning framework, DeepMicroFinder, which integrated the neural network and transfer learning [7]. DeepMicroFinder is advantageous in overcoming the regional effects of gut microbes and achieving cross‐regional diagnosis of T2D with high accuracy, as well as identifying reliable disease‐related microbial markers.

## 1 RESULTS AND DISCUSSION

### The framework of DeepMicroFinder

**FIGURE 1:**
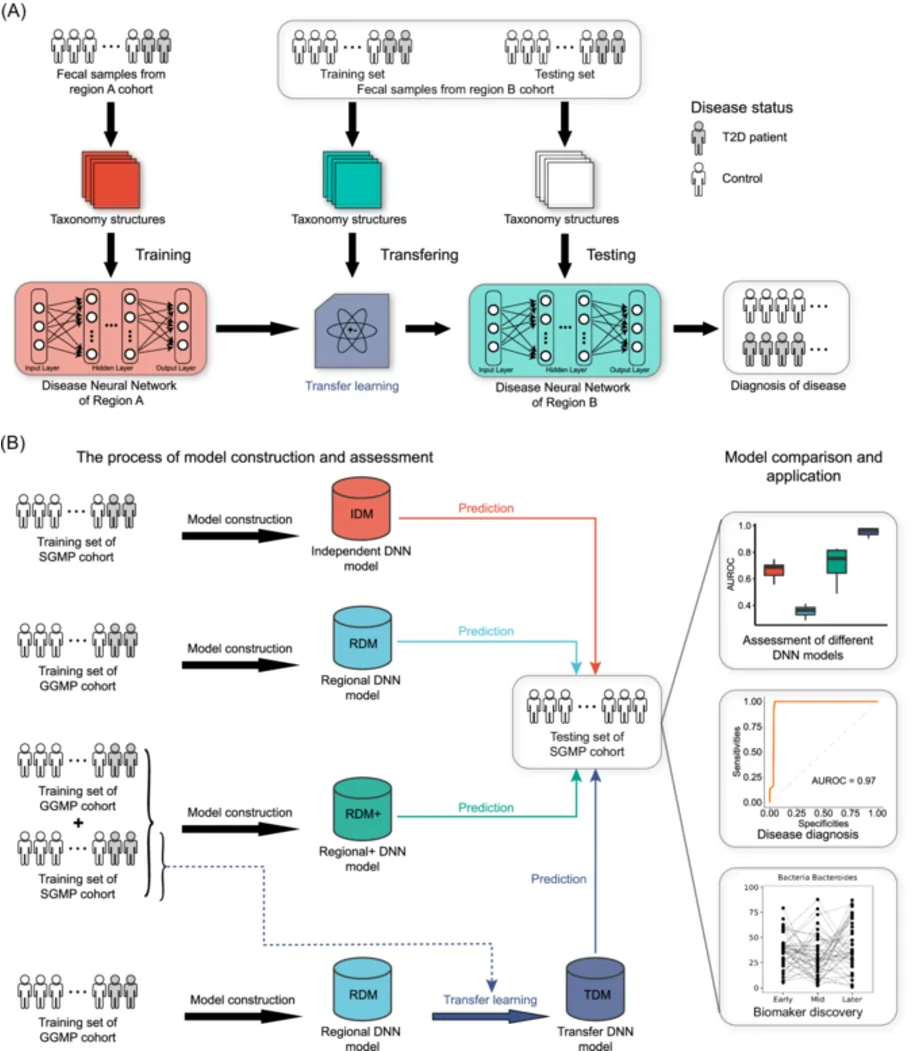
The rationale and workflow of DeepMicroFinder. (A) The process of model construction and transfer learning for cross‐regional diagnosis of diseases. (B) The experiment design and workflow. Samples of each cohort were randomly divided into the training subset and the testing subset, and four models were constructed for assessment: (1) Independent disease neural network (DNN) model: ab initio training the DNN model on the training subset and testing on the testing subset of SGMP cohort, respectively. (2) Regional DNN model: ab initio training the DNN model using the training subset of the Guangdong Gut Microbiome Project (GGMP) cohort and testing it on the testing subset of the Shandong Gut Microbiome Project (SGMP) cohort. (3) Regional+ DNN model: ab initio training the DNN model using the training subset of the GGMP cohort as well as the training subset of the SGMP cohort, then testing it on the testing subset of the SGMP cohort. (4) Transfer DNN model: ab initio training the DNN model using the training subset of the GGMP cohort, followed by applying transfer learning to a certain proportion (from 20% to 80%) of samples from the SGMP cohort to generate the transfer DNN model, and then testing the transfer DNN model on the testing subset of the SGMP cohort. The three boxes on the right represent the evaluation and applications of DeepMicroFinder, including cross‐regional diagnosis of T2D and biomarker discovery.

DeepMicroFinder utilizes the taxonomic structures and abundance tables of the microbial communities as inputting data to ab initio training the disease neural network (DNN) models, and diagnoses diseases based on differences in gut microbial community composition and structure between cases and controls (Figure 1A). Notably, DeepMicroFinder could exceed regional limitations gut microbiome through the transfer learning algorithms: By using the gut microbial profiles of a certain proportion of a cohort from another region for transfer learning, the existing DNN model undergoes structural and parametric adjustments to generate a transfer DNN model, enabling accurate disease diagnosis in the target region.

To assess the framework of DeepMicroFinder, we obtained the genus‐level taxonomy profiles of 2603 samples from the Guangdong Gut Microbiome Project (GGMP, including 604 T2D patients and 1999 controls) [5] and 700 samples from the Shandong Gut Microbiome Project (SGMP, including 614 T2D patients and 86 controls). Four DNN models (including the independent DNN model, regional DNN model, regional + DNN model, and transfer DNN model) were constructed based on these two cohorts (Figure 1B). For a fair assessment, we compared the performance of the four models for cross‐regional diagnosis of T2D, respectively (performance measures see Supplementary Methods). Then we utilized DeepMicroFinder to identify the microbial markers that were region‐specific or effective in the dietary fiber intervention therapy.

### Transfer learning exceeds the limitations of regional effects on the cross‐regional diagnosis of T2D

The gut microbial communities of the participants from GGMP and SGMP exhibited significant heterogeneity. At the phylum level, Tenericutes were more prevalent in GGMP, whereas Bacteroidetes dominated in SGMP (Figure S1A). Both alpha‐diversity and beta‐diversity indicated significant differences in the gut microbiome between samples from these two cohorts (Figures S1B,C and S2). The regional heterogeneity may limit the applicability of traditional machine learning models in disease diagnosis.

Then, we assessed the effectiveness of DeepMicroFinder on the cross‐regional diagnosis of T2D (Figure 1B). Four DNN models were evaluated on the SGMP testing subset for T2D diagnosis accuracy. The benchmark results have shown that the regional DNN model had the lowest area under the receiver operating characteristic curve (AUROC, average AUROC = 0.365) when the proportion of the testing subset was 80%, while the average AUROC of the independent DNN model was 0.544 (Figure S3). Notably, the regional+ DNN model, constructed based on the samples from GGMP and the training subset from SGMP, had a higher average AUROC (0.679) compared to the independent DNN model. Interestingly, the transfer DNN model had the highest average AUROC (0.680) (Figure S3), suggesting that transfer learning could overcome the regional effects limiting traditional machine learning methods in the cross‐regional diagnosis of T2D. Notably, the average AUROC of the transfer DNN model increased with the increase of the sample size of the training subset (Figure S3). When the training subset comprised 80% of samples, the average AUROC of the transfer DNN model reached 0.957, indicating its profound ability to diagnose T2D.

### Identification of region‐specific and T2D‐related microbial biomarkers

Given the best performance of the transfer DNN model, we next used the “Leave‐One‐Out” method (see Supplementary Methods) based on the transfer DNN model to identify four classes of microbial signatures: region‐specific and T2D‐related microbes, region‐shared and T2D‐related microbes, region‐specific and T2D‐unrelated microbes, as well as region‐shared and T2D‐unrelated microbes (Table 1). The region‐specific and T2D‐related microbes, such as *Delftia*, *Prevotellaceae*, and *Lactobacillaceae*, are likely associated with variations in T2D development across different regions. The exposure of *Delftia* may increase susceptibility to chronic inflammation in patients with type 1 diabetes [8], and the abundance of *Prevotellaceae* significantly increased in a women's cohort with gestational diabetes mellitus [9]. Further exploration of these microbes is beneficial for explaining the complex pathology of T2D and leads to personalized therapies for patients in different regions. *Turicibacter*, *Ruminococcus*, and *Prevotella* were identified as region‐shared and T2D‐related microbes, among which *Ruminococcus* was reported to be associated with the occurrence of nonalcoholic fatty liver disease in T2D diabetic patients [10]. These microbes had universal correlations with T2D in different regions, indicating that broad‐spectrum therapeutics of T2D can be developed for these common microbes in the future.

**TABLE 1:** The microbial markers identified by the transfer disease neural network (DNN) model.

Group	T2D‐related		T2D‐unrelated	
	Region‐specific	Region‐shared	Region‐specific	Region‐shared
**Microbial markers (genus level)**	*Delftia*	*Turicibacter*	*Aeromonas*	*Pelomonas*
	*Prevotellaceae*	*Ruminococcus*	*Proteus*	*Anaerobiospirillum*
	*Lactobacillaceae*	*Prevotella*	*Succinivibrio*	*Streptococcaceae*
	*Alkanindiges*	*Lachnospiraceae*	*S24‐7*	*Oxalobacteraceae*
	*Anaerostipes*	*Bacillus*	*Shigella*	*Aggregatibacter*
		*Alistipes*	*TM7‐3*	*[Barnesiellaceae]*
		*[Eubacterium]*	*Sutterella*	*Pseudoxanthomonas*
			*Achromobacter*	*Aeromonadales*
			*Comamonas*	*Actinomyces*
			*Epulopiscium*	*Cronobacter*
			*Abiotrophia*	*Klebsiella*
			*Kocuria*	*Butyricicoccus*
			*Gemellaceae*	*Massilia*
			*Desulfovibrio*	
			*Actinobacillus*	

*Note*: Region‐specific and region‐shared microbial markers were screened out according to their contributions to the transfer disease neural network (DNN) model. The top 20 microbial markers with the highest contribution were identified as region‐specific, while the 20 microbial markers with the lowest contribution were identified as region‐shared. Pearson correlation analysis was performed between all microbial markers and the status of type 2 diabetes (T2D). The microbial markers with a person correlation value greater than 0.1 or less than −0.1 and a *p*‐value less than 0.05 were considered T2D‐related, and the other microbial markers were considered T2D‐unrelated.

### Trajectory analysis of the dietary fiber intervention cohort

**FIGURE 2:**
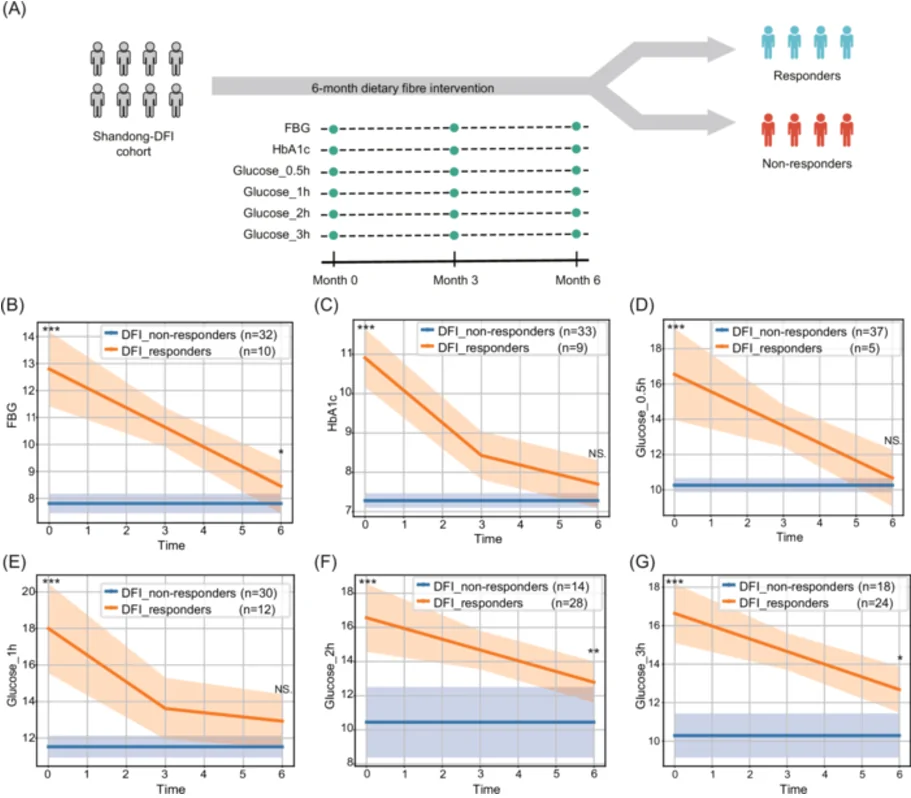
Trajectory analysis and baseline characteristic comparison of population with dietary fiber intervention. (A) Schematic diagram of population with dietary fiber intervention. (B–G) Trajectory plot of Group‐based Trajectory Model with fasting blood glucose (FBG), hemoglobin A1c (HbA1c) 0.5 h glucose, 1 h glucose, 2 h glucose, and 3 h glucose as the outcome variable. The *x*‐axis depicts the duration of dietary fiber intervention, and the *y*‐axis represents the corresponding outcome values. The solid line illustrates the trajectory of the estimated average outcome values, demonstrating their change over the course of the dietary fiber intervention. The shaded portion represents the 95% confidence intervals of the estimated average values, providing a visual representation of the uncertainty associated with the model's estimates. *p < 0.05; **p < 0.01; ***p < 0.005; Mann–Whitney *U* test.

Dietary fiber intervention has been widely used in the clinical T2D treatment [6]. It is plausible that gut microbial markers associated with T2D are significantly influenced by this dietary fiber intervention, therefore, identifying such biomarkers could provide broader insights into the mechanisms behind how dietary fiber intervention affects host gut microbes. For further investigating DeepMicroFinder's capability in identifying microbial biomarkers during the disease progression and exploring the impact of dietary fiber on the gut microbiota and clinical indicators of T2D patients, we introduced another cohort from Shandong province, Shandong dietary fiber intervention cohort (Shandong‐DFI), including 42 T2D patients who received dietary fiber intervention treatment, and each patient was collected fecal samples and clinical indicators measurements at three different time points (including early, mid, and later intervention stages), and a total of 250 fecal samples were collected and performed 16 s rRNA gene sequencing (Figure 2A).

Six essential glycemic‐related indicators: fasting blood glucose (FBG), hemoglobin A1c (HbA1c), 0.5 h glucose, 1 h glucose, 2 h glucose, and 3 h glucose, had been chosen as the primary variables for conducting trajectory analysis respectively (Figure 2A). The Group‐based Trajectory Models analysis demonstrated a significant division of the dietary fiber intervention population into two distinct subgroups, characterized by contrasting developmental trends for each characteristic (Tables S1–S13). These subgroups were identified as DFI_non‐responders and DFI_responders, with Average Posterior Probabilities (Avepp) of group assignment exceeding 80% (Table S14), indicating a high degree of heterogeneity within the population.

The DFI_responders group had markedly higher levels of fasting blood glucose, glycosylated hemoglobin, and postprandial glucose compared to the DFI_non‐responders group at baseline (Figure 2B–G). However, following DFI, the significance of differences in FBG and postprandial glucose levels at 2 and 3 h between the two groups was notably reduced, while the other glycemic indicators no longer exhibited noteworthy differences. (Figure 2B–G). This stage‐sensitive phenomenon is highly related to the baseline characteristics of these features in the dietary intervention group, which indicates that dietary fiber intervention has the potential to significantly enhance symptom management among high‐risk individuals with Type 2 Diabetes. Due to variations in the grouping criteria, the number of responders and non‐responders differed across indicators. To comprehensively assess the intervention's effectiveness, we incorporated clinical expertise and combined the analysis results using a union approach. Specifically, a patient was classified as a responder if they exhibited a significant change in at least one of the six indicators. Finally, 29 out of 42 individuals, who were classified as DFI_responders, showed a noticeable improvement in symptoms following DFI, resulting in a response rate of 69%.

### Stage‐specific correlations between microbial markers and clinical indicators

We then measured the dynamic changes in the relative abundance of microbial markers in the DFI_responders group. One hundred two genera were shared by three cohorts (GGMP, SGMP, and Shandong‐DFI) (Figure S4A). These microbes were ranked according to their contributions to the cross‐regional diagnosis of T2D of the transfer DNN model (Figure S4B). There were significant changes in the relative abundance of the top 20 important‐ranked microbes in the DFI_responders group at different DFI stages (Figure S4C). Several genera, including *Shigella*, *Sutterella*, and *Achromobacter*, were reported to be associated with the occurrence or treatment of T2D [11, 12].

We next analyzed the correlations between the microbial markers and the patients' clinical indicators of the DFI_responders group. We calculated the Spearman correlations between the top 40 important‐ranked microbial markers (based on their relative abundance) identified by the transfer DNN model and the 28 clinical indicators (based on their relative content) in the whole process and three stages of dietary fiber intervention, respectively (Figures S5A and S6). A number of microbial markers were associated with the clinical indicators across the entire dietary fiber intervention process, part of which had a significant change in the relative abundance, such as *Shigella*, *Sutterella*, *Achromobacter*, *Comamonas*, *Anaerostipes*, and *Clostridium* (Figures S4C and S5A).

Notably, we noticed that the correlations between microbial markers and clinical indicators were stage‐specific. For instance, *Shigella* was positively correlated with the number of leukocytes across the whole dietary fiber intervention, while no correlation was found in the early and middle stages, and a negative correlation was found in the later stage (Figures S5A and S6). The relative abundance of *Shigella* decreased in the DFI_responders group after the dietary fiber intervention, indicating its significant role in the treatment of T2D (Figure S4C). Intriguingly, dietary fiber is associated with the telomere length of leukocytes and may lead to an increase in the number of leukocytes, and the vacuoles of polymorphonuclear leukocytes could trap and efficiently kill *Shigella* [13, 14, 15, 16], these previous results rationalized the association between Shigella and leukocytes, supported by subsequent findings of increased leukocytes. Moreover, the average relative abundance of *Sutterella* in the DFI_responders group increased after the dietary fiber intervention (Figure S4C), and it was positively correlated with albumin and fasting insulin and negatively correlated with platelet across the entire dietary fiber intervention process (Figure S5A).

We further explored the changes in the clinical indicators in the DFI_responders group. The 28 clinical indicators were divided into three groups, including a significantly increased group, a significantly decreased group, and a nonsignificant change group (Figures S5B and S5C). With the progress of the DFI process, the levels of Leukocyte, Lymphocyte, Triglyceride (TG), Creatinine [17], and Hemoglobin (Hb) increased significantly, while the levels of aspartate aminotransferase‐to‐alanine aminotransferase ratio (AST/ALT ratio), Free fatty acids, high‐density lipoprotein (HDL), Alanine aminotransferase, FBG, and HbA1c [18] decreased significantly. The changes observed in clinical indicators aligned with previous studies demonstrating elevated fasting blood sugar in T2D patients, which decreases following dietary fiber intervention (Figure S5C) [19]. Interestingly, indicators related to blood lipids (Free fatty acids, HDL) and liver function (AST/ALT ratio, Alanine aminotransferase) also dropped significantly, reflecting that DFI not only reduces blood sugar but also regulates liver function and blood lipids. The alterations in clinical indicators suggest that, despite individual variations, the DFI_responders group exhibited improved clinical indicators, potentially attributable to the impact of dietary fiber intervention on the gut microbiota of these patients.

We admit that there are still limitations in this study. Although three independent cohorts were used in this study, confounding factors, such as age and gender of the participants, were not removed. Future studies should include more diverse samples and remove the influence of these confounding factors. Besides, only the top‐level network was frozen during the transfer learning process; further optimization is required to achieve more flexible parameters and structure freezing. Moreover, the pre‐training mode that uses pre‐encoded microbial signatures might result in the loss of valuable microbial signatures, and more microbial characteristics for pre‐training models and scalable general‐purpose model construction will be necessary in future work.

## 2 CONCLUSION

We proposed the deep learning framework, DeepMicroFinder, to exceed the regional effects and realize the cross‐regional diagnosis of T2D, identified region‐specific and T2D‐related microbial biomarkers, discovered the pivotal microbial biomarkers affected by dietary fiber intervention, and confirmed their correlations with the clinical indicators of T2D patients. Broadly, DeepMicroFinder exemplifies transfer learning's potential in microbiome and clinical medicine, suggesting the role of artificial intelligence in breaking through clinical bottlenecks.

## AUTHOR CONTRIBUTIONS

**Qunye Zhang**: Writing—review and editing; conceptualization. **Nan wang**: Methodology; writing—original draft; writing—review and editing; visualization; formal analysis; software; data curation. **Fanghua Zhang**: Resources; investigation. **Bin Chen**: Methodology; investigation; writing—original draft; writing—review and editing; visualization; data curation. **Yihui Wang**: Methodology; visualization; writing—original draft; writing—review and editing. **Zhongchao Wang**: Resources; investigation. **Changying Zhao**: Investigation. **Chuandi Jin**: Investigation. **Dashuang Sheng**: Investigation; formal analysis. **Kaile Yue**: Formal analysis; investigation. **Daifeng Jiang**: Investigation. **Liaomei Gao**: Resources; investigation. **Haohong Zhang**: Resources; investigation. **Zixin Kang**: Formal analysis. **Mingyue Cheng**: Formal analysis. **Xiaoli Ma**: Resources; investigation. **Haiyan Wang**: Resources; investigation. **Dongming Hu**: Resources; investigation. **Jun Wang**: Resources; investigation. **Yuantao Liu**: Resources; investigation. **Chenhong Zhou**: Resources; investigation. **Minxiu Yao**: Resources; investigation. **Guoping Zhao**: Conceptualization. **Yangang Wang**: Conceptualization; investigation; resources. **Zhe Wang**: Conceptualization; resources. **Kang Ning**: Conceptualization; writing—original draft; writing—review and editing; methodology. **Lei Zhang**: Conceptualization; writing—review and editing; writing—original draft; project administration; supervision; funding acquisition.

## CONFLICT OF INTEREST STATEMENT

The authors declare no conflicts of interest.

## 1 ETHICS STATEMENT

This study was approved by the Ethics Committee of The Affiliated Hospital of Qingdao University (QYFY WZLL 25763) and was performed in accordance with the principles of the Helsinki Declaration. The Shandong‐DFI cohort trial is registered with the Chinese Clinical Trial Registry (ChiCTR) under the number ChiCTR‐ONC‐16009323.

## Supporting information

Supplementary Material.

## Data Availability

The data that support the findings of this study are available from the corresponding author upon reasonable request. Raw 16s rRNA gene (V3‐V4 region) sequencing data of fecal samples from GGMP are downloaded in the European Bioinformatics Institute (EBI) database of European Molecular Biology Laboratory (EBI accession number PRJEB18535) at https://www.ebi.ac.uk/ena/browser/view/PRJEB18535. Samples from the Shandong cohort (V1‐V2 region) are available at the National Omics Data Encyclopedia (NODE) with the accession numbers OEP00000124 and OEP00000125 (https://www.biosino.org/node/project/detail/OEP00000124, https://www.biosino.org/node/project/detail/OEP00000125). The data and scripts used are saved in https://github.com/HUST-NingKang-Lab/DeepMicroFinder. Supplementary materials (methods, figures, tables, graphical abstract, slides, videos, Chinese translated version, and update materials) may be found in the online DOI or iMeta Science http://www.imeta.science/imetaomics/.
