# Supplementary material for: Transfer learning identifies bacterial signatures for cross‐regional diagnosis of type 2 diabetes and enable stage‐sensitive dietary fiber intervention
Source: IMetaOmics. 2025 May 4;2(2):e70021. doi: 10.1002/imo2.70021 (PMC12806279; doi:10.1002/imo2.70021)
Supplement: Supplementary file 1 — Supplementary Material. [file IMO2-2-e70021-s001.docx]

**Supporting information to**

**Transfer learning identifies bacterial signatures for cross-regional diagnosis of Type 2 Diabetes and enable stage-sensitive dietary fiber intervention**

Qunye Zhang^1#^, Nan Wang^2#^, Fanghua Zhang^3#^, Bin Chen^1#^, Yihui Wang^1#^, Zhongchao Wang^4^, Changying Zhao^1^, Chuandi Jin^1^, Dashuang Sheng^1^, Kaile Yue^1^, Daifeng Jiang^5^, Liaomei Gao^6^, Haohong Zhang^2^, Zixin Kang^2^, Mingyue Cheng^2^, Xiaoli Ma^7^, Haiyan Wang^8^, Dongming Hu^4^, Jun Wang^4^, Yuantao Liu^9^, Chenhong Zhou^7^, Minxiu Yao^3^, Guoping Zhao^1, 10, 11*^, Yangang Wang^4*^, Zhe Wang^12*^, Kang Ning^2*^, Lei Zhang^1, 3, 10*^

^1^Microbiome-X, School of Public Health; Department of Cardiology, Qilu Hospital; Cheeloo College of Medicine, Shandong University, Jinan, 250012, China

^2^Key Laboratory of Molecular Biophysics of the Ministry of Education, Hubei Key Laboratory of Bioinformatics and Molecular-imaging, Center of AI Biology, Department of Bioinformatics and Systems Biology, College of Life Science and Technology, Huazhong University of Science and Technology, Wuhan, 430074, China

^3^Department of Endocrinology, Qingdao Central Hospital, University of Health and Rehabilitation Sciences (Qingdao Central Hospital), Qingdao, 266011, China

^4^Qingdao University Medical Group: Department of Endocrinology and Metabolism, The Affiliated Hospital of Qingdao University; Department of Endocrinology and Metabolism, The Third People's Hospital of Qingdao, Qingdao, 266041, China

^5^Shandong Academy of Sciences Science and Technology Services Platform (Shandong Academy of Sciences Foreign Students Pioneer Park), Jinan, 250014, China

^6^Shandong Provincial Maternal and Child Health Care Hospital Affiliated to Qingdao University, Jinan, 250014, China

^7^Department of Endocrinology, Qingdao Municipal Hospital (Group), Qingdao, 266011, China

^8^Department of Endocrinology, The Eighth People's Hospital of Qingdao, Qingdao, 266100, China

^9^Department of Endocrinology, Qilu Hospital of Shandong University (Qingdao), No.758 Hefei Road, Shibei District, Qingdao, 266035, China

^10^State Key Laboratory of Microbial Technology, Shandong University, Qingdao, 266237, China

^11^CAS Key Laboratory of Computational Biology, Bio-Med Big Data Center, Shanghai Institute of Nutrition and Health, University of Chinese Academy of Sciences, Chinese Academy of Sciences, Shanghai, 200031, China

^12^Department of Endocrinology & Geriatrics, Shandong Provincial Hospital Affiliated to Shandong First Medical University, Jinan, 250001, China.

^#^These authors contributed equally: Qunye Zhang, Nan Wang, Fanghua Zhang, Bin Chen, Yihui Wang.

^*^Correspondence: [zhanglei7@sdu.edu.cn](mailto:zhanglei7@sdu.edu.cn) (Lei Zhang); ningkang@hust.edu.cn (Kang Ning); wangzhe@sdfmu.edu.cn (Zhe Wang); [wangyg1966@126.com](mailto:wangyg1966@126.com) (YangangWang); gpzhao@sibs.ac.cn (Guoping Zhao)

# Supplementary methods

## Participants and sample collection

This study was approved by the Ethics Committee of The Affiliated Hospital of Qingdao University (QYFY WZLL 25763) and was performed in accordance with the principles of the Helsinki Declaration. From 2015 to 2018, 614 patients that clinically diagnosed as T2DM in Qingdao Central Hospital, The Affiliated Hospital of Qingdao University, and Shandong Provincial Hospital Affiliated to Shandong First Medical University were randomly recruited and enrolled into the Shandong Gut Microbiome Project (SGMP). The Shandong dietary fiber intervention cohort (Shandong-DFI) trial is registered with the Chinese Clinical Trial Registry (ChiCTR) under the number ChiCTR-ONC-16009323. A total of 126 fecal samples were collected from 42 T2DM patients before, during (at month 3), and after dietary fiber intervention (at month 6) at Qingdao Central Hospital. All participants provided their informed consent for the study.

A total of 86 individuals (no reported diseases, fasting blood glucose (FBG) < 6.1 and BMI < 24) were brought into SGMP that were regarded as healthy controls. The diagnostic criteria of T2DM were according to the 1999 WHO diagnostic criteria for diabetes, and in accordance with one of the following conditions: (a) random blood glucose (venous plasma glucose) ≥ 11.1 mmol/ L; (b) fasting plasma glucose (zero calorie intake for at least 8 h) ≥ 7.0 mmol/L; (c) oral glucose tolerance test, 2 h venous plasma glucose ≥ 11.1 mmol/L. Subjects with the following conditions were excluded: < 18 years of age; pregnant or lactating women; with other systemic diseases (e.g., lung, cardiovascular, gastrointestinal, liver, and kidney diseases); taking antibiotics, steroids or probiotics in the previous 3 months; varied dietary habits (e.g., vegan diet); no evidence of untreated or uncontrolled caries or periodontal disease, precancerous or cancerous oral lesions, and oral candidiasis, after dental examination. Guangdong Gut Microbiome Project (GGMP) is the largest Eastern population-based gut microbiome dataset that covering 7009 individuals in South China, in which, 604 patients with T2DM (self-report T2DM or FBG ≥ 7.0 mmol/L) and 1999 healthy controls confirmed with the same criteria with that in SGMP were brought into this present study as well. All these participants have no antibiotic use within 1 month before donating a fecal sample. Fecal samples were collected at local hospitals and transported to local laboratories for processing within 10 min. 250 mg stool was preserved in sterilized 2 mL tubes containing pure ethanol, aliquoted and stored at -80 °C for 16S rRNA sequencing.

## DNA extraction and 16S rRNA gene sequencing

Total genome DNA was extracted from the fecal samples using the improved CTAB (cetyl trimethylammonium bromide) method. DNA from stool was extracted by 2 × CTAB (cetyltrimethyl ammonium bromide), phenol chloroform mixture (phenol:chloroform:isoamyl alcohol = 25:24:1). In the later steps of DNA isolation, we used the spin column from SanPrep Column DNA Gel Extraction Kit (Sangon Biotech, China) to purify and recover the DNA rapidly. Concentration and quality of DNA were determined by a NanoDrop 2000 spectrophotometer (Thermo Scientific, USA). The V1 and V2 variable region of the 16S rRNA gene was amplified by PCR and the primer sequences were 27F (5’-AGAGTTTGATCMTGGCTCAG-3’) and 338R(5’-GCTGCCTCCCGTAGGAGT-3’) modified with specific barcodes. QIAquick PCR Purification Kit (Qiagen) were used to purify PCR products. An equimolar library was constructed by pooling samples, and the resulting library was sequenced on the HiSeq 2500 (Illumina).

## Raw data processing and analysis

Raw 16s rRNA gene (V3-V4 region) sequencing data of fecal samples from GGMP are downloaded in the European Bioinformatics Institute (EBI) database of European Molecular Biology Laboratory (EBI accession number PRJEB18535) at <https://www.ebi.ac.uk/ena/browser/view/PRJEB18535>. Samples from Shandong cohort (V1-V2 region) are available at the National Omics Data Encyclopedia (NODE) with the accession number OEP00000124 and OEP00000125(<https://www.biosino.org/node/project/detail/OEP00000124>, https://www.biosino.org/node/project/detail/OEP00000125).

The 16s rRNA gene sequencing data of GGMP, SGMP, Shandong-DFI cohort was manipulated by using Quantitative Insights Into Microbial Ecology 2 (QIIME2, version 2020.2). Briefly, raw reads were demultiplexed, joined, and denoised (deblur algorithm) to generate amplicon sequence variants (ASVs) and representative sequences. Samples with sequence depth < 5000 were excluded. Taxonomy of the ASVs was assigned by using the GreenGene database (version 13.8) classifier with 99% similarity.

## The construction of the disease neural network model

The forms of the inputting data were the microbial abundance table and the metadata of the samples’ disease status. First, a mapping relationship between the taxonomic profiles and the phylogenetic tree was established based on the inputting data, generating a regular abundance matrix, then it was standardized and converted into a relative abundance matrix. Then, the relative abundance matrix was standardized by Z-score to become a standard abundance matrix. The modeling process of the disease neural network (DNN) was based on the standard abundance matrix. The DNN consisted of four modules: (1) the “base” module is to obtain the features of the inputting standard abundance matrix on the low level, (2) the “inter” module, which contains three Dense NN layers, is to obtain the features of different hierarchy layers, (3) the “integ” module, which contains a concatenation NN layer and a Dense NN layer, is to integrate the features of different hierarchy layers, and (4) the “output” module, which contains a Dense NN layer, is to estimate the probability of disease or health status according to the integrated representations of different hierarchy layers. During forward propagation, the representation of each lower layer is integrated into the corresponding higher layer using multiple “integ” modules, which establish the initialization parameters between neural network layers. Backward propagation involves optimizing the parameters of the entire model. This is achieved through the utilization of gradient descent coupled with the backpropagation algorithm, enabling the solution of the model’s parameters.

## Transfer learning process

The existing model could be divided into bottom- and top-level nodes. The bottom- and top-level nodes could be represented as

$$\Theta=\{\theta_{bottom},\theta_{top}\}$$

The bottom-level nodes have the potential to be applied in emerging datasets, while the top-level nodes can only be applied to the existing datasets. The format of the microbial abundance table of the emerging datasets is $X_{new}\in R_{n\times d}$, in which $n$ represents the number of samples and $d$ represents the number of taxonomy structures, together with $Y_{new}$ which represents the ontology structure of the emerging datasets.

The transfer learning process involves three steps for effective knowledge transfer. Firstly, the lower-level nodes are locked to ensure their exclusion from the transfer process as following

$$\theta_{bottom}^{'}\leftarrow\theta_{bottom}$$

then the new community structure is encoded and introduced, accompanied by modifications to the structure and weights between the higher-level nodes

$$\theta_{top}^{'}\leftarrow\theta_{top}+\Delta\theta_{top}$$

in which $\Delta\theta_{top}$ was generated by the encoded new community structure of the emerging datasets. This process, referred to as “Transfer”, aims to facilitate the incorporation of microbial data from the new community.

Secondly, the forward and backward algorithms are then applied iteratively to update the parameters of higher-level nodes until convergence is achieved.

Forward propagation:

$$\hat{Y}_{new}=f\left( X_{new};\theta_{top} \right)=\sigma\left( \theta_{n}^{'}\cdot\sigma\left( \theta_{n-1}^{'}\cdot\cdots\sigma\left( \theta_{3}^{'}\cdot\sigma\left( \theta_{2}^{'}\cdot\sigma\left( \theta_{1}^{'}\cdot X_{new} \right) \right) \right) \right) \right)$$

in which $\sigma$ represents activation function (ReLU, tanh, and sigmoid) corresponding to each layer of $\theta_{top}$, and $n$ represents the number of layers of $\theta_{top}$.

Backward propagation:

$$\theta_{k}^{'}\leftarrow\theta_{k}^{'}-\eta\nabla_{\theta_{k}^{'}}\mathcal{L}\left( \theta_{top};X_{new},Y_{new} \right) \left( k=1,2,3,4,\cdots,n \right)$$

$\mathcal{L}$represents the multitask loss function as following

$$\mathcal{L=}\sum_{l=1}^{L} \lambda_{l}\cdot\mathrm{CrossEntropy}\left( \hat{Y}_{new}^{(l)},Y_{new}^{(l)} \right)$$

in which $L$ represents the number of layers and $\lambda$represents the weight of each layer. This step is referred to as the “Fast Adaptation” process.

In the final stage, the bottom-level nodes are unlocked and updated iteratively using the new microbial data and the training of backward algorithms

$$\theta_{k}^{'}\leftarrow\theta_{k}^{'}-\eta\nabla_{\theta_{k}^{'}}\mathcal{L}\left( \theta_{top};X_{new},Y_{new} \right) \left( k=1,2,3,4,\cdots,m \right)$$

in which $m$ represents the number of layers of $\theta_{bottom}$. This phase is referred to as “Fine-tuning”. The transfer step trains model with 1e-3 learning rate, and we used an early stopping callback to monitor the validation loss with 15 stop patience.

## Performance measures

For the area under the receiver operating characteristic (AUROC) curve computation, we set the threshold from 0 to 1 with a step size of 0.01. The result of the logical operation is 1 if the contribution of the node is greater than the threshold, else 0. We calculated True Positive, True Negative, False Positive, and False Negative for calculating True Positive Rate and False Positive Rate at every threshold, and then we obtained the AUC curve. By calculating the area under the AUC curve as AUROC. Each node represents an ecological classification of a community.

## Leave-One-Out method for biomarker discovery

Each of the microbial features of the SGMP cohort was removed in turn when constructing the transfer DNN model, and then the absolute value of the change of the AUROC of the transfer DNN model was calculated. For each microbial feature, the removing procedure was repeated five times, and the mean value of the absolute value of the change of the AUROC was used as the contribution of the microbial feature to the transfer DNN model. Microbial features with the strongest change of the AUROC (top 20) would be designated as “region-specific”, on the contrary, microbial features with the least change of the AUROC (top 20) would be designated as “region-shared”.

## Correlation analysis

The correlation between the microbial markers of SGMP and T2D was calculated by Spearman correlation. The input matrix of each microbial marker was the relative abundance table, and the input matrix of disease information was represented as disease was 1 and control was 0.

The correlation between the microbial markers and metabolites of patients receiving dietary fiber intervention was calculated by Pearson correlation. The input matrix of each microbial marker was the relative abundance table, and the input matrix of each metabolite was the content in the body.

## Trajectory analysis

Trajectory analysis of the dietary fiber intervention (DFI) population was conducted using Stata 15.1 software to fit the Group-based Trajectory Models. To compare the baseline characteristics between the responders and non_responders groups, Mann-Whitney *U* test was employed.

## Codes availability

The pre-trained models and source codes of scripts for training, querying and transfer learning are made publicly available at <https://github.com/HUST-NingKang-Lab/DeepMicroFinder>. The program “EXPERT” is available at <https://github.com/HUST-NingKang-Lab/EXPERT>.

# Supplementary figures


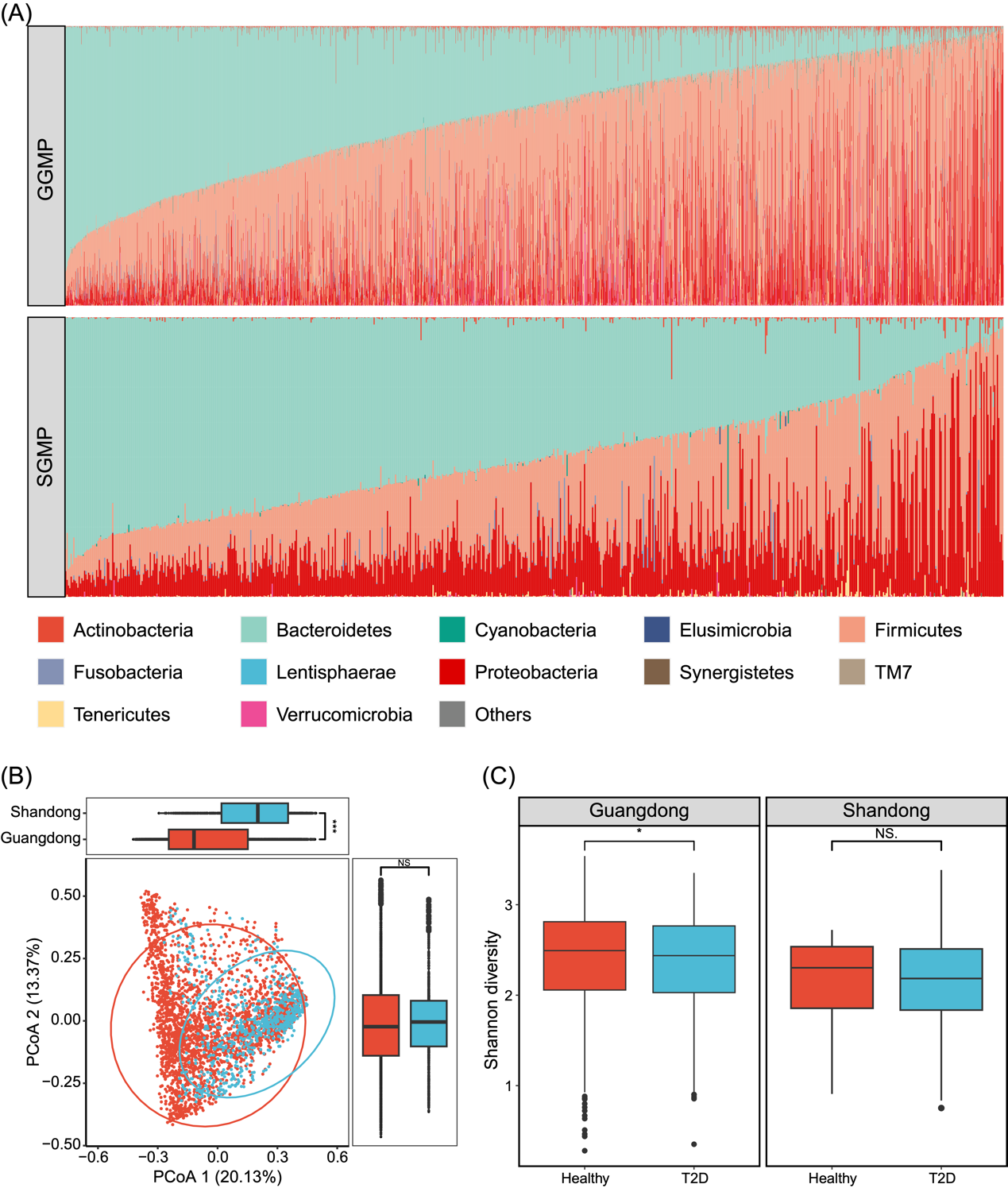


## Fig S1 Heterogeneity in gut microbes of the individuals from different regions.

(A) The microbial composition of Guangdong Gut Microbiome Project (GGMP) samples and Shandong Gut Microbiome Project (SGMP) samples at the phylum level. The stack plot shows the top 10 phyla with the highest abundance, and the rest were divided into others. (B) Principal coordinates analysis (PCoA) plots of GGMP samples and SGMP samples. The dots represent 2,063 samples from GGMP and 700 samples from SGMP. Differences in beta-diversity and beta-dispersion among locations were tested by Bray-Curtis distance. The ellipse represents a 95% confidence interval. (C) The comparison of microbial community diversity between healthy and type 2 diabetes (T2D) groups of GMMP and SGMP cohorts. The Shannon index represents alpha diversity. ^*^*p* < 0.05; ^**^*p* < 0.01; ^***^*p* < 0.005; NS; no significance; Mann-Whitney *U* test.

**
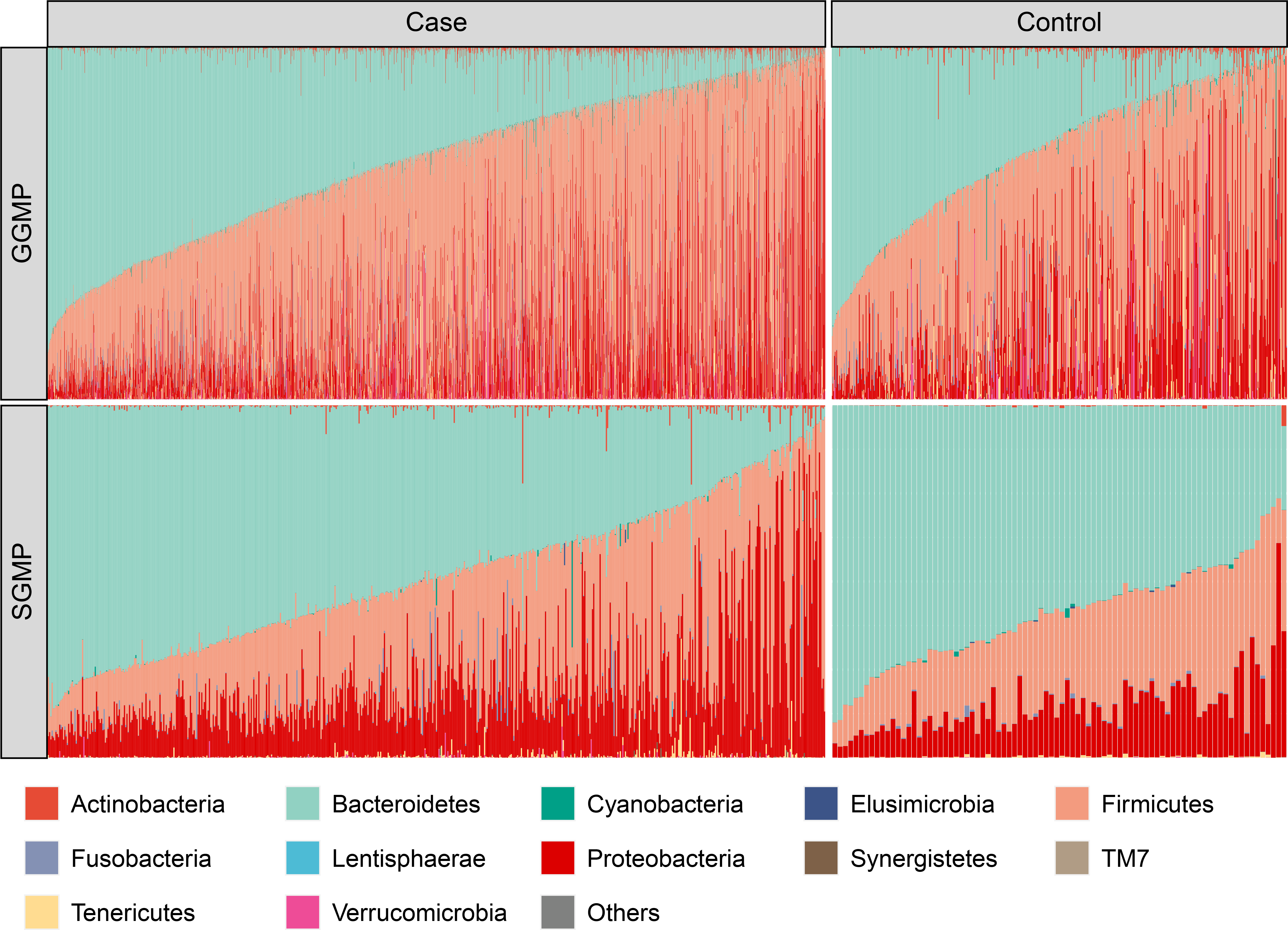
**

## Fig S2 The microbial composition of different groups of Guangdong Gut Microbiome Project (GGMP) and Shandong Gut Microbiome Project (SGMP) samples at the phylum level

The stack bar plots show the microbial composition of different groups of GGMP and SGMP samples at the phylum level. The top 10 phyla with the highest abundance are shown in the figure, and the rest were divided into others.


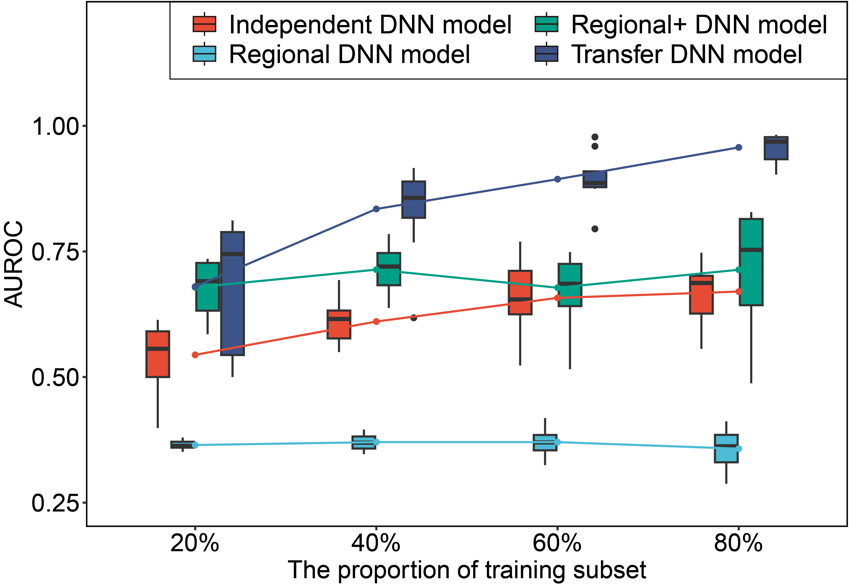


## Fig S3 The gradient evaluation of different disease neural network (DNN) models.

The percentages on the X-axis represent the proportion of the training subset from the Shandong Gut Microbiome Project (SGMP) cohort, and the Y-axis shows the values of the area under the receiver operating characteristic (AUROC). The boxplots show the AUROC values of the disease neural network (DNN) models for diagnosing T2D, the construction and evaluation of each DNN model was repeated 10 times. The lines show the change in average AUROC of four models with sample size increasing and the points represent the average AUROC of each model. For all the boxplots, boxes represent the interquartile range between the first and third quartiles and the line inside represents the median. Whiskers denote the lowest and highest values within the 1.5 × interquartile range (IQR) from the first and third quartiles, respectively.


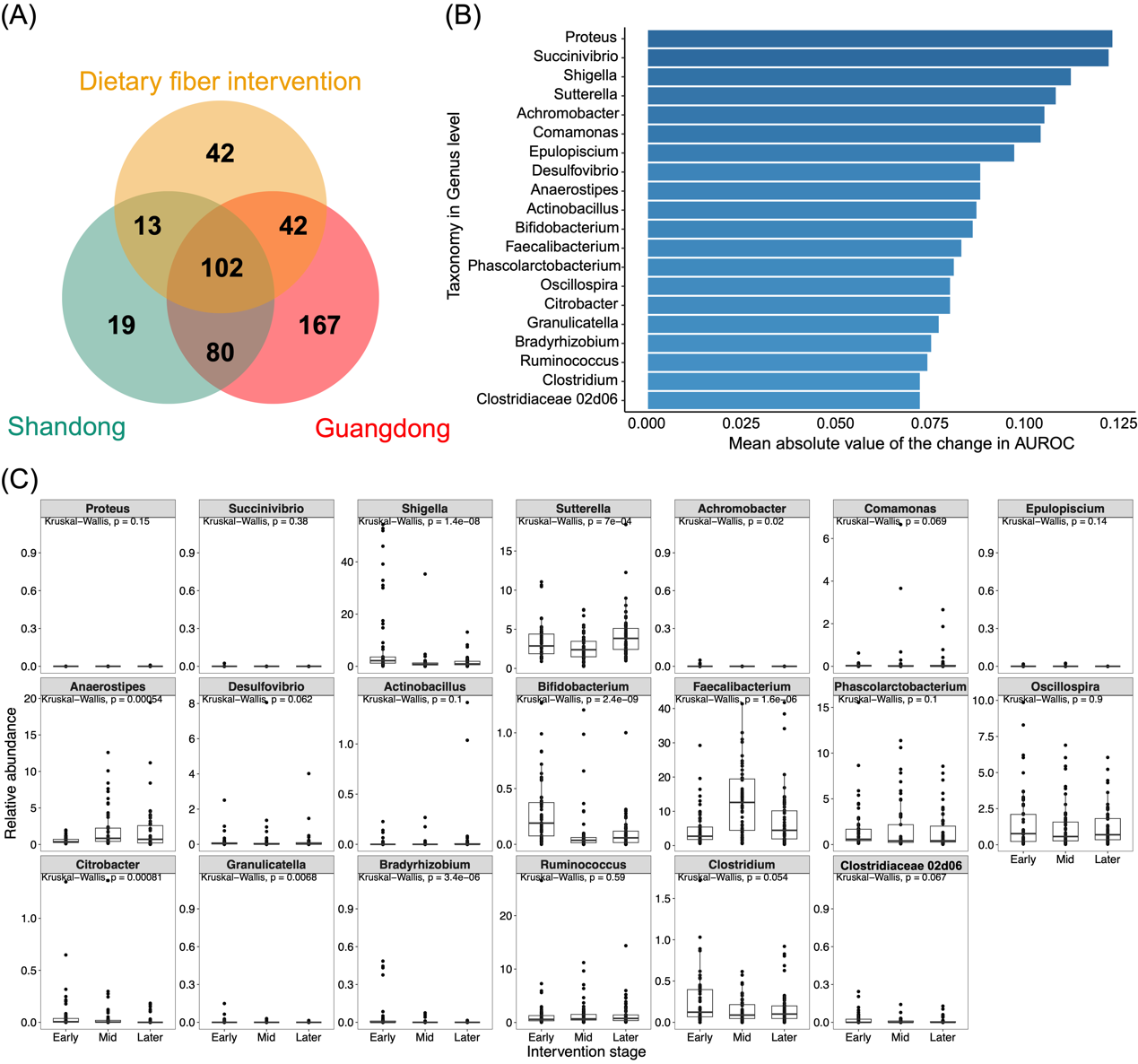


## Fig S4 Transfer learning is advantageous in detecting microbial biomarkers.

(A) The number of genera shared by three cohorts. (B) The top 20 genera with the highest contribution in the cross-regional diagnosis of type 2 diabetes (T2D) of the transfer disease neural network (DNN) model. The X-axis represents the changes in the AUROC of the transfer DNN model after using the "Leave-One-Out" method to remove a genus from the abundance table. (C) Changes in the relative abundance of the top 20 genera at different dietary intervention stages. Statistical significance was calculated by Kruskal-Wallis test.

**
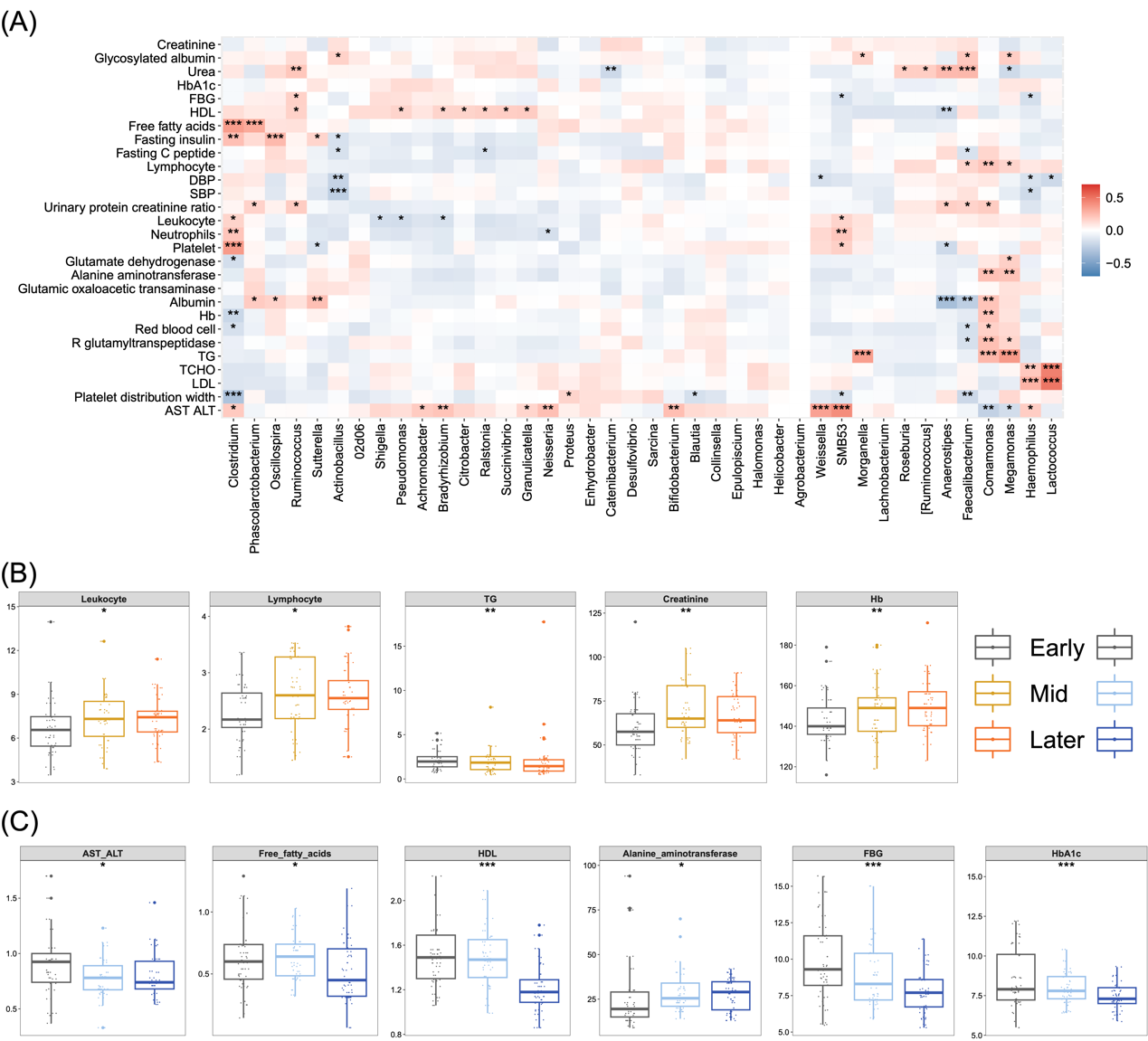
**

## Fig S5 Correlation between microbial markers and clinical indicators during dietary fiber intervention.

(A) Association in the whole process of dietary fiber intervention between clinical indicators and microbial biomarkers. The X-axis represents the microbial markers, and the Y-axis represents the clinical indicators. ^*^*p* < 0.05; ^**^*p* < 0.01; ^***^*p* < 0.005. (B) Clinical indicators with significantly elevated levels during dietary fiber intervention. (C) Clinical indicators with significantly decreased levels during dietary fiber intervention. For all the boxplots, boxes represent the IQR between the first and third quartiles and the line inside represents the median. Whiskers denote the lowest and highest values within the 1.5 × interquartile range (IQR) from the first and third quartiles, respectively. ^*^*p* < 0.05; ^**^*p* < 0.01; ^***^*p* < 0.005; Kruskal-Wallis test.


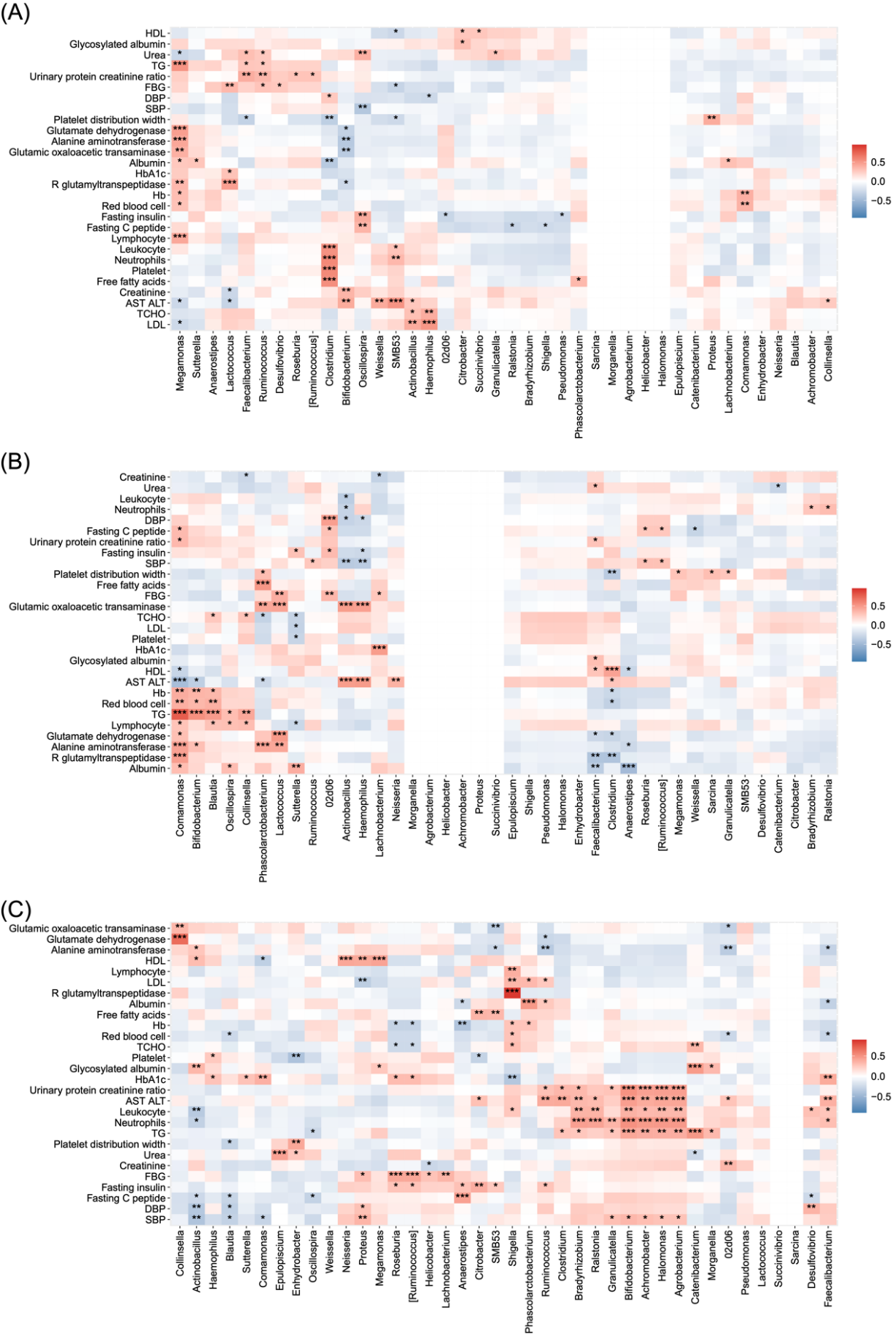


## Fig S6 Association in the three stages (early (A), mid (B), and later (C)) of dietary fiber intervention between clinical indicators and microbial biomarkers

The X-axis represents the microbial markers, and the Y-axis represents the clinical indicators. ^*^*p* < 0.05; ^**^*p* < 0.01; ^***^*p* < 0.005.

# Supplementary tables

**Table S1** Evaluation of Group-based Trajectory Model (GBTM) with Fasting Blood Glucose (FBG) as the Outcome Variable

| Maximum Likelihood Estimate | | | | |
| --- | --- | --- | --- | --- |
| Model: Censored Normal (cnorm) | | | | |
| Group Parameter | Estimate | Standard Error | T for H0:Parameter=0 | Prob > T |
| 1 Intercept | 7.80899 | 0.18493 | 42.227 | <0.0001 |
| 2 Intercept | 12.82222 | 0.58028 | 22.096 | <0.0001 |
| Linear | -0.72869 | 0.13748 | -5.3 | <0.0001 |
|  | | | | |
| Sigma | 1.65682 | 0.11354 | 14.593 | <0.0001 |
| Group membership |  | | | |
| 1 (%) | 76.93106 | 7.28791 | 10.556 | <0.0001 |
| 2 (%) | 23.06894 | 7.28791 | 3.165 | 0.0019 |

Group 1: dietary fiber intervention non-responders (DFI_non-responders); Group 2: dietary fiber intervention responders (DFI_responders)

**Table S2** Trajectory Prediction of Group Assignment of Group-based Trajectory Model (GBTM) with Fasting Blood Glucose (FBG) as the Outcome Variable

| Patient_id | Group | ProbG1 | ProbG2 |
| --- | --- | --- | --- |
| 12 | 2 | 0.0157042 | 0.9842958 |
| 13 | 1 | 0.9717613 | 0.0282386 |
| 14 | 1 | 0.9969012 | 0.0030988 |
| 15 | 1 | 0.9999935 | 6.50E-06 |
| 16 | 1 | 0.9999995 | 4.47E-07 |
| 17 | 1 | 0.9972533 | 0.0027467 |
| 18 | 1 | 0.9999518 | 0.0000482 |
| 19 | 1 | 0.9999504 | 0.0000496 |
| 22 | 1 | 0.9999815 | 0.0000185 |
| 23 | 1 | 0.9903103 | 0.0096897 |
| 24 | 1 | 0.9690818 | 0.0309182 |
| 25 | 2 | 0.0270437 | 0.9729563 |
| 27 | 2 | 0.1702922 | 0.8297077 |
| 28 | 2 | 0.0012344 | 0.9987656 |
| 30 | 1 | 0.9764603 | 0.0235397 |
| 31 | 1 | 0.8517128 | 0.1482872 |
| 32 | 1 | 0.9954171 | 0.0045829 |
| 33 | 1 | 0.9999651 | 0.000035 |
| 34 | 1 | 0.9714135 | 0.0285865 |
| 35 | 1 | 0.9749268 | 0.0250732 |
| 36 | 2 | 0.0000665 | 0.9999335 |
| 37 | 1 | 0.9979795 | 0.0020205 |
| 38 | 1 | 0.9990217 | 0.0009783 |
| 39 | 1 | 0.984069 | 0.0159309 |
| 40 | 1 | 0.9998941 | 0.0001059 |
| 42 | 1 | 0.9666424 | 0.0333576 |
| 43 | 1 | 0.9999978 | 2.19E-06 |
| 45 | 2 | 0.0376097 | 0.9623903 |
| 46 | 1 | 0.9878148 | 0.0121852 |
| 47 | 1 | 0.9973862 | 0.0026138 |
| 48 | 1 | 0.9997607 | 0.0002393 |
| 49 | 2 | 0.1668545 | 0.8331455 |
| 50 | 1 | 0.9998024 | 0.0001977 |
| 51 | 1 | 0.9999784 | 0.0000216 |
| 52 | 1 | 0.9999989 | 1.15E-06 |
| 54 | 2 | 0.2815301 | 0.7184699 |
| 55 | 2 | 6.73E-06 | 0.9999933 |
| 56 | 1 | 0.9983881 | 0.0016119 |
| 57 | 1 | 0.9999418 | 0.0000582 |
| 58 | 2 | 0.0014779 | 0.9985222 |
| 59 | 1 | 0.9947848 | 0.0052152 |
| 60 | 1 | 0.988677 | 0.011323 |

Group 1: dietary fiber intervention non-responders (DFI_non-responders); Group 2: dietary fiber intervention responders (DFI_responders); ProG1: the probability that an individual is classified as a DFI_non-responder; ProG2: the probability that an individual is classified as a DFI_responder

**Table S3** Evaluation of Group-based Trajectory Model (GBTM) with Hemoglobin A1c (HbA1c) as the Outcome Variable

| Maximum Likelihood Estimate | | | | |
| --- | --- | --- | --- | --- |
| Model: Censored Normal (cnorm) | | | | |
| Group Parameter | Estimate | Standard Error | T for H0:Parameter=0 | Prob > T |
| 1 Intercept | 7.28387 | 0.09602 | 75.859 | <0.0001 |
| 2 Intercept | 0.33696 |  | 32.38 | <0.0001 |
| Linear | -1.11822 | 0.27333 | -4.091 | 0.0001 |
| Quadratic | 0.09729 | 0.04307 | 2.259 | 0.0256 |
|  | | | | |
| Sigma | 0.91066 | 0.06008 | 15.157 | <0.0001 |
| Group membership |  | | | |
| 1 (%) | 78.31014 | 6.63723 | 11.799 | <0.0001 |
| 2 (%) | 21.68986 | 6.63723 | 3.268 | 0.0014 |

Group 1: dietary fiber intervention non-responders (DFI_non-responders); Group 2: dietary fiber intervention responders (DFI_responders)

**Table S4** Trajectory Prediction of Group Assignment of Group-based Trajectory Model (GBTM) with Hemoglobin A1c (HbA1c) as the Outcome Variable

| Patient_id | Group | ProbG1 | ProbG2 |
| --- | --- | --- | --- |
| 12 | 1 | 0.9989977 | 0.0010023 |
| 13 | 1 | 0.9977022 | 0.0022978 |
| 14 | 1 | 0.9996384 | 0.0003616 |
| 15 | 1 | 0.9999816 | 0.0000184 |
| 16 | 1 | 0.9999999 | 3.15E-08 |
| 17 | 1 | 0.9999938 | 6.22E-06 |
| 18 | 1 | 0.9999898 | 0.0000102 |
| 19 | 1 | 0.9985687 | 0.0014313 |
| 22 | 2 | 0.0001865 | 0.9998135 |
| 23 | 1 | 0.9999923 | 7.77E-06 |
| 24 | 1 | 0.9999999 | 4.07E-08 |
| 25 | 2 | 0.0108456 | 0.9891545 |
| 27 | 2 | 0.0628415 | 0.9371585 |
| 28 | 2 | 1.82E-06 | 0.9999982 |
| 30 | 1 | 0.979507 | 0.020493 |
| 31 | 2 | 0.0029178 | 0.9970822 |
| 32 | 1 | 0.9999461 | 0.0000539 |
| 33 | 1 | 0.9995548 | 0.0004452 |
| 34 | 1 | 0.9999478 | 0.0000521 |
| 35 | 1 | 0.9900974 | 0.0099026 |
| 36 | 1 | 0.9653989 | 0.0346011 |
| 37 | 1 | 0.9999953 | 4.74E-06 |
| 38 | 2 | 0.0003711 | 0.9996288 |
| 39 | 1 | 0.9986598 | 0.0013402 |
| 40 | 1 | 0.9999999 | 3.59E-08 |
| 42 | 1 | 0.9999933 | 6.74E-06 |
| 43 | 1 | 1 | 6.23E-10 |
| 45 | 2 | 7.36E-07 | 0.9999993 |
| 46 | 1 | 0.9999532 | 0.0000468 |
| 47 | 1 | 0.9999953 | 4.71E-06 |
| 48 | 1 | 0.9999956 | 4.32E-06 |
| 49 | 1 | 0.8970153 | 0.1029847 |
| 50 | 1 | 0.9997773 | 0.0002227 |
| 51 | 1 | 0.9998074 | 0.0001926 |
| 52 | 1 | 1 | 9.46E-09 |
| 54 | 1 | 0.982897 | 0.017103 |
| 55 | 2 | 0.0057768 | 0.9942232 |
| 56 | 1 | 0.9999987 | 1.26E-06 |
| 57 | 1 | 0.9994308 | 0.0005692 |
| 58 | 2 | 0.0005079 | 0.999492 |
| 59 | 1 | 0.9999751 | 0.0000249 |
| 60 | 1 | 0.9999987 | 1.34E-06 |

Group 1: dietary fiber intervention non-responders (DFI_non-responders); Group 2: dietary fiber intervention responders (DFI_responders); ProG1: the probability that an individual is classified as a DFI_non-responder; ProG2: the probability that an individual is classified as a DFI_responder

**Table S5** Evaluation of Group-based Trajectory Model (GBTM) with Glucose_0.5h as the Outcome Variable

| Maximum Likelihood Estimate | | | | |
| --- | --- | --- | --- | --- |
| Model: Censored Normal (cnorm) | | | | |
| Group Parameter | Estimate | Standard Error | T for H0:Parameter=0 | Prob > T |
| 1 Intercept | 10.25923 | 0.20544 | 49.938 | <0.0001 |
| 2 Intercept | 16.59009 | 1.04116 | 15.934 | <0.0001 |
| Linear | -0.98741 | 0.24227 | -4.076 | 0.0001 |
|  | | | | |
| Sigma | 1.99687 | 0.13305 | 15.008 | <0.0001 |
| Group membership |  | | | |
| 1 (%) | 87.30196 | 5.86093 | 14.896 | <0.0001 |
| 2 (%) | 12.69804 | 5.86093 | 2.167 | 0.0322 |

Group 1: dietary fiber intervention non-responders (DFI_non-responders); Group 2: dietary fiber intervention responders (DFI_responders)

**Table S6** Trajectory Prediction of Group Assignment of Group-based Trajectory Model (GBTM) with Glucose_0.5h as the Outcome Variable

| Patient_id | Group | ProbG1 | ProbG2 |
| --- | --- | --- | --- |
| 12 | 1 | 0.978098 | 0.021902 |
| 13 | 1 | 0.996248 | 0.003752 |
| 14 | 1 | 0.999955 | 4.56E-05 |
| 15 | 1 | 0.999753 | 0.000247 |
| 16 | 1 | 0.999999 | 6.55E-07 |
| 17 | 1 | 0.999795 | 0.000205 |
| 18 | 1 | 0.999902 | 0.000098 |
| 19 | 1 | 0.99983 | 0.00017 |
| 22 | 1 | 1 | 3.95E-08 |
| 23 | 1 | 0.999786 | 0.000214 |
| 24 | 1 | 0.995835 | 0.004165 |
| 25 | 2 | 0.123749 | 0.876251 |
| 27 | 1 | 0.992768 | 0.007232 |
| 28 | 2 | 0.044848 | 0.955152 |
| 30 | 1 | 0.999668 | 0.000332 |
| 31 | 1 | 0.999667 | 0.000333 |
| 32 | 1 | 0.996737 | 0.003264 |
| 33 | 1 | 0.999966 | 3.41E-05 |
| 34 | 1 | 0.999584 | 0.000416 |
| 35 | 1 | 0.999269 | 0.000731 |
| 36 | 2 | 5.97E-05 | 0.99994 |
| 37 | 1 | 0.813476 | 0.186524 |
| 38 | 1 | 0.999865 | 0.000135 |
| 39 | 1 | 0.999128 | 0.000872 |
| 40 | 1 | 0.999846 | 0.000154 |
| 42 | 1 | 0.998785 | 0.001215 |
| 43 | 1 | 0.999999 | 8.35E-07 |
| 45 | 1 | 0.986535 | 0.013465 |
| 46 | 1 | 0.974056 | 0.025944 |
| 47 | 1 | 0.997864 | 0.002136 |
| 48 | 1 | 1 | 5.08E-07 |
| 49 | 1 | 0.847233 | 0.152767 |
| 50 | 1 | 0.999675 | 0.000325 |
| 51 | 1 | 0.999964 | 3.58E-05 |
| 52 | 1 | 1 | 5.71E-09 |
| 54 | 1 | 0.993815 | 0.006185 |
| 55 | 2 | 0.002502 | 0.997498 |
| 56 | 1 | 0.999992 | 7.78E-06 |
| 57 | 1 | 0.983705 | 0.016295 |
| 58 | 2 | 0.005959 | 0.994041 |
| 59 | 1 | 0.941787 | 0.058213 |
| 60 | 1 | 0.997115 | 0.002885 |

Group 1: dietary fiber intervention non-responders (DFI_non-responders); Group 2: dietary fiber intervention responders (DFI_responders); ProG1: the probability that an individual is classified as a DFI_non-responder; ProG2: the probability that an individual is classified as a DFI_responder

**Table S7** Evaluation of Group-based Trajectory Model (GBTM) with Glucose_1h as the Outcome Variable

| Maximum Likelihood Estimate | | | | |
| --- | --- | --- | --- | --- |
| Model: Censored Normal (cnorm) | | | | |
| Group Parameter | Estimate | Standard Error | T for H0:Parameter=0 | Prob > T |
| 1 Intercept | 11.53408 | 0.30219 | 38.168 | <0.0001 |
| 2 Intercept | 18.04332 | 0.97634 | 18.481 | <0.0001 |
| Linear | -2.09487 | 0.6414 | -3.266 | 0.0014 |
| Quadratic | 0.20697 | 0.10229 | 2.023 | 0.0452 |
|  | | | | |
| Sigma | 2.34559 | 0.16316 | 14.376 | <0.0001 |
| Group membership |  | | | |
| 1 (%) | 72.42515 | 9.48878 | 7.633 | <0.0001 |
| 2 (%) | 27.57485 | 9.48878 | 2.906 | 0.0043 |

Group 1: dietary fiber intervention non-responders (DFI_non-responders); Group 2: dietary fiber intervention responders (DFI_responders);

**Table S8** Trajectory Prediction of Group Assignment of Group-based Trajectory Model (GBTM) with Glucose_1h as the Outcome Variable

| Patient_id | Group | ProbG1 | ProbG2 |
| --- | --- | --- | --- |
| 12 | 1 | 0.771056 | 0.228944 |
| 13 | 2 | 0.329556 | 0.670444 |
| 14 | 1 | 0.973507 | 0.026493 |
| 15 | 1 | 0.991952 | 0.008049 |
| 16 | 1 | 0.999946 | 5.36E-05 |
| 17 | 1 | 0.998551 | 0.001449 |
| 18 | 1 | 0.984924 | 0.015076 |
| 19 | 1 | 0.999424 | 0.000576 |
| 22 | 1 | 0.999098 | 0.000902 |
| 23 | 1 | 0.999309 | 0.000691 |
| 24 | 1 | 0.946247 | 0.053753 |
| 25 | 2 | 0.020512 | 0.979489 |
| 27 | 2 | 0.220568 | 0.779432 |
| 28 | 2 | 0.000572 | 0.999428 |
| 30 | 1 | 0.98405 | 0.01595 |
| 31 | 1 | 0.813319 | 0.186681 |
| 32 | 1 | 0.995016 | 0.004984 |
| 33 | 1 | 0.998425 | 0.001575 |
| 34 | 1 | 0.951949 | 0.048051 |
| 35 | 1 | 0.991398 | 0.008602 |
| 36 | 2 | 0.006226 | 0.993774 |
| 37 | 2 | 0.000112 | 0.999888 |
| 38 | 1 | 0.993368 | 0.006632 |
| 39 | 2 | 0.450143 | 0.549857 |
| 40 | 1 | 0.988449 | 0.011551 |
| 42 | 1 | 0.998616 | 0.001384 |
| 43 | 1 | 0.999913 | 8.73E-05 |
| 45 | 2 | 0.013016 | 0.986984 |
| 46 | 1 | 0.976896 | 0.023104 |
| 47 | 1 | 0.990337 | 0.009663 |
| 48 | 1 | 0.999951 | 4.87E-05 |
| 49 | 1 | 0.528988 | 0.471012 |
| 50 | 1 | 0.995967 | 0.004033 |
| 51 | 1 | 0.99461 | 0.00539 |
| 52 | 1 | 1 | 2.56E-07 |
| 54 | 1 | 0.893075 | 0.106925 |
| 55 | 2 | 0.038625 | 0.961375 |
| 56 | 1 | 0.999946 | 5.36E-05 |
| 57 | 2 | 0.138093 | 0.861907 |
| 58 | 2 | 0.002515 | 0.997485 |
| 59 | 2 | 0.474177 | 0.525823 |
| 60 | 1 | 0.966151 | 0.03385 |

Group 1: dietary fiber intervention non-responders (DFI_non-responders); Group 2: dietary fiber intervention responders (DFI_responders); ProG1: the probability that an individual is classified as a DFI_non-responder; ProG2: the probability that an individual is classified as a DFI_responder

**Table S9** Evaluation of Group-based Trajectory Model (GBTM) with Glucose_2h as the Outcome Variable

| Maximum Likelihood Estimate | | | | |
| --- | --- | --- | --- | --- |
| Model: Censored Normal (cnorm) | | | | |
| Group Parameter | Estimate | Standard Error | T for H0:Parameter=0 | Prob > T |
| 1 Intercept | 10.44564 | 1.04403 | 10.005 | <0.0001 |
| 2 Intercept | 16.57615 | 0.9214 | 17.99 | <0.0001 |
| Linear | -0.63032 | 0.18154 | -3.472 | 0.0007 |
|  | | | | |
| Sigma | 3.12163 | 0.21794 | 14.324 | <0.0001 |
| Group membership |  | | | |
| 1 (%) | 32.65391 | 15.62393 | 2.09 | 0.0386 |
| 2 (%) | 67.34609 | 15.62393 | 4.31 | <0.0001 |

Group 1: dietary fiber intervention non-responders (DFI_non-responders); Group 2: dietary fiber intervention responders (DFI_responders)

**Table S10** Trajectory Prediction of Group Assignment of Group-based Trajectory Model (GBTM) with Glucose_2h as the Outcome Variable

| Patient_id | Group | ProbG1 | ProbG2 |
| --- | --- | --- | --- |
| 12 | 2 | 0.112387 | 0.887613 |
| 13 | 2 | 0.019226 | 0.980774 |
| 14 | 2 | 0.325714 | 0.674286 |
| 15 | 2 | 0.014334 | 0.985666 |
| 16 | 1 | 0.998196 | 0.001804 |
| 17 | 1 | 0.563704 | 0.436296 |
| 18 | 2 | 0.113709 | 0.886291 |
| 19 | 1 | 0.943677 | 0.056323 |
| 22 | 1 | 0.587491 | 0.412509 |
| 23 | 1 | 0.647254 | 0.352746 |
| 24 | 2 | 0.177698 | 0.822302 |
| 25 | 2 | 0.001709 | 0.998291 |
| 27 | 2 | 0.001744 | 0.998256 |
| 28 | 2 | 0.001472 | 0.998528 |
| 30 | 2 | 0.154256 | 0.845744 |
| 31 | 2 | 0.130812 | 0.869188 |
| 32 | 1 | 0.793473 | 0.206527 |
| 33 | 2 | 0.135071 | 0.864929 |
| 34 | 2 | 0.017913 | 0.982087 |
| 35 | 2 | 0.1556 | 0.8444 |
| 36 | 2 | 0.007379 | 0.992621 |
| 37 | 2 | 0.000249 | 0.999751 |
| 38 | 2 | 0.136348 | 0.863652 |
| 39 | 2 | 0.014734 | 0.985266 |
| 40 | 2 | 0.467445 | 0.532555 |
| 42 | 1 | 0.910958 | 0.089042 |
| 43 | 1 | 0.993236 | 0.006764 |
| 45 | 2 | 0.004383 | 0.995617 |
| 46 | 2 | 0.143318 | 0.856682 |
| 47 | 1 | 0.512788 | 0.487213 |
| 48 | 1 | 0.965345 | 0.034655 |
| 49 | 2 | 0.002338 | 0.997662 |
| 50 | 1 | 0.518807 | 0.481193 |
| 51 | 1 | 0.995673 | 0.004327 |
| 52 | 1 | 0.998931 | 0.001069 |
| 54 | 2 | 0.018467 | 0.981533 |
| 55 | 2 | 0.002429 | 0.997571 |
| 56 | 1 | 0.967346 | 0.032654 |
| 57 | 2 | 0.000165 | 0.999835 |
| 58 | 2 | 0.000441 | 0.999559 |
| 59 | 2 | 0.000609 | 0.999391 |
| 60 | 2 | 0.157804 | 0.842196 |

Group 1: dietary fiber intervention non-responders (DFI_non-responders); Group 2: dietary fiber intervention responders (DFI_responders); ProG1: the probability that an individual is classified as a DFI_non-responder; ProG2: the probability that an individual is classified as a DFI_responder

**Table S11** Evaluation of Group-based Trajectory Model (GBTM) with Glucose_3h as the Outcome Variable

| Maximum Likelihood Estimate | | | | |
| --- | --- | --- | --- | --- |
| Model: Censored Normal (cnorm) | | | | |
| Group Parameter | Estimate | Standard Error | T for H0:Parameter=0 | Prob > T |
| 1 Intercept | 10.29441 | 0.58006 | 17.747 | <0.0001 |
| 2 Intercept | 16.64387 | 0.73121 | 22.762 | <0.0001 |
| Linear | -0.66095 | 0.15527 | -4.257 | <0.0001 |
|  | | | | |
| Sigma | 2.90336 | 0.20487 | 14.172 | <0.0001 |
| Group membership |  | | | |
| 1 (%) | 40.49264 | 11.21237 | 3.611 | 0.0004 |
| 2 (%) | 59.50736 | 11.21237 | 5.307 | <0.0001 |

Group 1: dietary fiber intervention non-responders (DFI_non-responders); Group 2: dietary fiber intervention responders (DFI_responders)

**Table S12** Trajectory Prediction of Group Assignment of Group-based Trajectory Model (GBTM) with Glucose_3h as the Outcome Variable

| Patient_id | Group | ProbG1 | ProbG2 |
| --- | --- | --- | --- |
| 12 | 2 | 0.255583 | 0.744417 |
| 13 | 2 | 0.044563 | 0.955437 |
| 14 | 1 | 0.591664 | 0.408336 |
| 15 | 2 | 0.00097 | 0.99903 |
| 16 | 1 | 0.999914 | 8.59E-05 |
| 17 | 1 | 0.588893 | 0.411108 |
| 18 | 2 | 0.449204 | 0.550796 |
| 19 | 1 | 0.984592 | 0.015408 |
| 22 | 1 | 0.885041 | 0.114959 |
| 23 | 1 | 0.864693 | 0.135307 |
| 24 | 2 | 0.130262 | 0.869738 |
| 25 | 2 | 0.001363 | 0.998637 |
| 27 | 2 | 0.000293 | 0.999707 |
| 28 | 2 | 0.021092 | 0.978908 |
| 30 | 1 | 0.53081 | 0.46919 |
| 31 | 2 | 0.210438 | 0.789562 |
| 32 | 1 | 0.93825 | 0.06175 |
| 33 | 1 | 0.889958 | 0.110042 |
| 34 | 2 | 0.008277 | 0.991723 |
| 35 | 2 | 0.058208 | 0.941792 |
| 36 | 2 | 0.003408 | 0.996593 |
| 37 | 2 | 0.018036 | 0.981964 |
| 38 | 2 | 0.006561 | 0.993439 |
| 39 | 2 | 0.019607 | 0.980394 |
| 40 | 1 | 0.807782 | 0.192218 |
| 42 | 1 | 0.976545 | 0.023455 |
| 43 | 1 | 0.999476 | 0.000524 |
| 45 | 2 | 0.000787 | 0.999213 |
| 46 | 2 | 0.037863 | 0.962138 |
| 47 | 1 | 0.661204 | 0.338796 |
| 48 | 1 | 0.995636 | 0.004365 |
| 49 | 2 | 0.002163 | 0.997837 |
| 50 | 1 | 0.804143 | 0.195857 |
| 51 | 1 | 0.998712 | 0.001288 |
| 52 | 1 | 0.999785 | 0.000215 |
| 54 | 2 | 0.084103 | 0.915897 |
| 55 | 2 | 0.001557 | 0.998443 |
| 56 | 1 | 0.989847 | 0.010153 |
| 57 | 2 | 0.000105 | 0.999895 |
| 58 | 2 | 0.000102 | 0.999898 |
| 59 | 2 | 0.006091 | 0.993909 |
| 60 | 2 | 0.139312 | 0.860688 |

Group 1: dietary fiber intervention non-responders (DFI_non-responders); Group 2: dietary fiber intervention responders (DFI_responders); ProG1: the probability that an individual is classified as a DFI_non-responder; ProG2: the probability that an individual is classified as a DFI_responder

**Table S13** Parameter Estimation of Group-based Trajectory Model (GBTM) Fit

| Variable | Time | Group | | | | | | | |
| --- | --- | --- | --- | --- | --- | --- | --- | --- | --- |
|  |  | DFI_non-responders | | | | DFI_responders | | | |
|  |  | Ave | Est | L95 | U95 | Ave | Est | L95 | U95 |
| FBG | 0 | 7.89 | 7.81 | 7.45 | 8.17 | 13 | 12.8 | 11.42 | 14.19 |
|  | 3 | 7.96 | 7.81 | 7.45 | 8.17 | 10.28 | 10.64 | 9.91 | 11.36 |
|  | 6 | 7.58 | 7.81 | 7.45 | 8.17 | 8.63 | 8.45 | 7.43 | 9.4 |
| HbA1c | 0 | 7.2 | 7.28 | 7.1 | 7.47 | 10.91 | 10.91 | 10.17 | 11.65 |
|  | 3 | 7.5 | 7.28 | 7.1 | 7.47 | 8.43 | 8.43 | 7.82 | 9.04 |
|  | 6 | 7.16 | 7.28 | 7.1 | 7.47 | 7.7 | 7.7 | 7.1 | 8.31 |
| Glucose_0.5h | 0 | 10.59 | 10.26 | 9.86 | 10.66 | 17.06 | 16.55 | 13.96 | 19.15 |
|  | 3 | 10.07 | 10.26 | 9.86 | 10.66 | 12.7 | 13.63 | 12.44 | 14.81 |
|  | 6 | 10.11 | 10.26 | 9.86 | 10.66 | 11.13 | 10.67 | 9.05 | 12.28 |
| Glucose_1h | 0 | 11.61 | 11.53 | 10.94 | 12.13 | 18.04 | 18 | 15.55 | 20.45 |
|  | 3 | 11.45 | 11.53 | 10.94 | 12.13 | 13.62 | 13.62 | 11.94 | 15.3 |
|  | 6 | 11.54 | 11.53 | 10.94 | 12.13 | 12.93 | 12.93 | 11.45 | 14.4 |
| Glucose_2h | 0 | 10.49 | 10.45 | 8.38 | 12.51 | 16.9 | 16.57 | 14.57 | 18.56 |
|  | 3 | 10.2 | 10.45 | 8.38 | 12.51 | 14.03 | 14.68 | 13.55 | 15.81 |
|  | 6 | 10.64 | 10.45 | 8.38 | 12.51 | 13.11 | 12.79 | 11.59 | 14 |
| Glucose_3h | 0 | 10.56 | 10.29 | 9.15 | 11.44 | 16.87 | 16.64 | 15.1 | 18.18 |
|  | 3 | 9.74 | 10.29 | 9.15 | 11.44 | 14.21 | 14.66 | 13.7 | 15.62 |
|  | 6 | 10.56 | 10.29 | 9.15 | 11.44 | 12.9 | 12.68 | 11.49 | 13.87 |

FBG: fasting blood glucose; hemoglobin A1c: HbA1c; Ave: data averages; Est: model estimates; L95: Lower bound of the 95% confidence interval; U95: Upper bound of the 95% confidence interval; DFI_non-responders: dietary fiber intervention non-responders; DFI_responders: dietary fiber intervention responders

**Table S14** Average Positerior Probabilities (Avepp) of Group Assignment and Bayesian Information Criterion (BIC) Statistics of Model Fit

| Variable | AvePP | | BIC  (*n* = 125) | BIC  (*n* = 42) |
| --- | --- | --- | --- | --- |
|  | DFI_non-responders | DFI_responders |  |  |
| FBG | 0.99 | 0.93 | -271.73 | -269.00 |
| HbA1c | 0.99 | 0.99 | -200.97 | -197.70 |
| Glucose_0.5h | 0.98 | 0.96 | -289.51 | -286.78 |
| Glucose_1h | 0.95 | 0.85 | -316.42 | -313.15 |
| Glucose_2h | 0.81 | 0.91 | -347.49 | -344.76 |
| Glucose_3h | 0.86 | 0.93 | -341.82 | -339.09 |

FBG: fasting blood glucose; hemoglobin A1c: HbA1c; DFI_non-responders: dietary fiber intervention non-responders; DFI_responders: dietary fiber intervention responders
